# Integrated analyses of microRNA-29 family and the related combination biomarkers demonstrate their widespread influence on risk, recurrence, metastasis and survival outcome in colorectal cancer

**DOI:** 10.1186/s12935-019-0907-x

**Published:** 2019-07-15

**Authors:** Qiliang Peng, Zhengyang Feng, Yi Shen, Jiahao Zhu, Li Zou, Yuntian Shen, Yaqun Zhu

**Affiliations:** 1 Department of Radiotherapy & Oncology, The Second Affiliated Hospital of Soochow University, San Xiang Road No. 1055, Suzhou, Jiangsu 215004 China; 2 Institute of Radiotherapy & Oncology, Soochow University, Suzhou, China; 3 Department of Oncology, The Second Affiliated Hospital of Soochow University, Suzhou, China; 4 Department of Radiation Oncology, The Affiliated Suzhou Science & Technology Town Hospital of Nanjing Medical University, Suzhou Science & Technology Town Hospital, Suzhou, China; 5 Tongda College of Nanjing University of Post and Telecommunications, Yangzhou, China

**Keywords:** Colorectal cancer, Biomarker, Meta-analysis, System biological analysis

## Abstract

**Background:**

Emerging evidence has revealed miR-29 family as promising biomarkers for colorectal cancer (CRC), but their biomarker potential and molecular mechanisms remain poorly understood.

**Methods:**

We performed a comprehensive meta-analysis to evaluate the biomarker performance of individual miR-29 and the related miRNA combination biomarkers. Meanwhile, we conducted an integrative bioinformatics analysis to unfold the underlying biological function of miR-29 and their relationship with CRC.

**Results:**

Using miR-29 expression to diagnose CRC produced 0.82 area under the curve, 70% sensitivity and 81% specificity while the combination biomarkers based on miR-29 enhanced the diagnostic power with an AUC of 0.86, a sensitivity of 78% and a specificity of 91%. For the prognosis evaluation, patients with higher expression of miR-29 had better survival outcome (pooled HR 0.78; 95% CI 0.56–1.07). In addition, miR-29 has also been identified as potential biomarker for predicting recurrence and metastasis in CRC. Then the genes regulated by the miR-29 family were retrieved and found closely associated with the molecular pathogenesis of CRC according to the gene ontology and pathway analysis. Furthermore, hub nodes and significant modules were identified from the protein–protein interaction network constructed with miR-29 family targets, which were also confirmed highly involved in the establishment and development of CRC.

**Conclusions:**

Current evidences suggest miR-29 family may become promising biomarkers for risk, recurrence, metastasis and survival outcome of CRC. Meanwhile our data highlight the potential clinical use of miRNA combination biomarkers. Nevertheless, further prospective studies are warranted before the application of the useful biomarkers in the clinical.

## Background

Colorectal cancer (CRC) is the third and second most commonly diagnosed cancers and the fourth and second most common causes of cancer related death respectively in men and women worldwide [1]. The clinical outcome strongly depends on tumor stage at presentation and the early diagnosis of CRC is associated with improved survival rates [2]. Nowadays, several early detection procedures of CRC have been established and are increasingly applied, including fecal occult-blood testing (FOBT), colonoscopy, and stool DNA test [3]. However, none of these methods has been developed as a optimal or universally accepted strategy due to their low adherence rates, high cost or low sensitivity [4]. Therefore, the development of novel non-invasive, sensitive, and specific diagnostic and prognostic biomarkers is highly demanded.

MicroRNAs (miRNAs) are a class of highly conserved single-stranded RNAs that epigenetically regulate protein expression at the post-transcriptional level [5]. Accumulating evidence has indicated that miRNAs are aberrantly expressed in various human cancers and crucial to tumorigenesis [6]. Acting as potential oncogenes or tumor suppressors in cancer initiation and development, miRNAs play vital roles in fundamental cellular processes including cell proliferation, apoptosis, differentiation, and migration [7]. Meanwhile, a number of studies in recent years have convincingly demonstrated that the expressions of various miRNAs are frequently dysregulated in CRC [8]. It is worth pointing out that miRNAs exhibit an outstanding stability in serum, plasma, urine, and other body fluids [9]. Considering the perfect biomarker features and critical involvement in the regulation of developmental, physiological and oncogenic processes, miRNAs show great promises to be convenient and informative for CRC diagnosis, prognosis and therapeutic efficacy.

Among all the reported miRNAs, microRNA-29 (miR-29) family, which consists of miR-29a, miR-29b, and miR-29c, has been considered to be related to aggressiveness and prognosis of malignant neoplasms and might function as promising biomarker for predicting the initiation, progression and pathogenesis of cancer [10]. Previous researches have demonstrated the availability of the members of miR-29 family in cancer diagnosis and prognosis [11]. Overwhelming evidence has indicated that aberrant expressions of the miR-29 family members are involved in the establishment and development of CRC [12]. Nevertheless, the biomarker applicability of miR-29 family of CRC remains controversial because of inconsistent results from different studies. Moreover, the molecular mechanism of miR-29 family in the tumorigenesis and cancer progression is still not very clear due to the present insufficient knowledge.

Herein, we summarized recent findings and discussed the potential value of miR-29 family as diagnostic and prognostic biomarkers for CRC. Different from traditional biomarker studies that focused on single molecule, we also explored the value of combination miRNA biomarkers based on miR-29 in CRC. Furthermore, we attempted to unfold the underlying biological function of miR-29 and their relationship with CRC through an integrative bioinformatics analysis.

## Materials and methods

### Publication search strategy

A comprehensive literature search of PubMed, Embase and Web of Science was performed, with the following keywords variably combined: “microRNA-29”, “miR-29”, “miRNA-29”, “cancer”, “carcinoma”, “tumor”, “neoplasm”, “colorectal”, “colon”, “rectal”, “rectum”, and “CRC”. The last search was done on 21 September 2018. We initially reviewed all articles by scanning the titles and abstracts to identify the relevant studies, and full texts were further perused for potentially eligible studies according to our including criteria. We also searched the references within the relevant review papers in order to identify other potentially eligible studies.

### Eligibility criteria

The eligible studies in this study must meet all the following criteria: (1) the diagnosis of CRC was made based on histopathological confirmation, (2) the associations between miR-29 expression and diagnostic or recurrence or metastasis or survival outcome were measured, and (3) studies directly provided true positive (TP), false positive (FP), false negative (FN), and true negative (TN) for diagnostic studies or hazard ratio (HR) and their 95% confidence intervals (CIs) for prognostic studies or with data available regarding these statistics.

Articles were excluded according to the following criteria: (1) reviews, editorials, letters, case reports, and conference abstracts, (2) duplicate publications, (3) unqualified data, and (4) non-English articles.

### Data extraction and quality assessment

Data extraction was independently conducted by two reviewers (Peng QL and Feng ZY). Data were extracted including study details (first author, published year, and country of publication), participants’ general features (number of patients, gender, age, and clinical stage), miRNA detection features (measurement methods and sample sources), and data needed for diagnostic meta-analysis (sensitivity, specificity, TP, FP, FN, TN and AUC) or prognostic meta-analysis (HR and their corresponding 95% CIs). Any discrepancy was resolved through discussion to reach a consensus. The quality of each study enrolled in the diagnostic meta-analysis was scored independently by two reviewers (Peng QL and Feng ZY) with the quality assessment of diagnostic accuracy studies 2 (QUADAS-2) [13]. For prognostic studies, the quality was assessed following the guidelines of the Newcastle–Ottawa Scale [14].

### Statistical analysis for meta-analysis

For the diagnostic meta-analyses, the bivariate meta-analysis model was selected to estimate the pooled sensitivity, specificity, positive likelihood ratio (PLR), negative likelihood ratio (NLR), and diagnostic odds ratio (DOR) with the corresponding 95% CIs [15]. The sensitivity and specificity of each eligible study were plotted to set up the summary receiver operating characteristic curve (SROC). Besides that, the area under the SROC curve (AUC) was calculated as quantitative measurements of the diagnostic accuracy [16]. For the prognostic meta-analyses, HRs and their 95% CIs were used to assess the impact of miR-29 expression on survival of CRC patients with a random-effect model if significant heterogeneity exists; otherwise, a fixed effect model was selected. In addition, heterogeneity of the pooled results was evaluated by the Q test and I^2^ [17]. A P-value less than 0.05 for the Q test or I^2^ larger than 50% suggests presence of heterogeneity. To explore the threshold effect, the Spearman correlation coefficient was employed. Subgroup, meta-regression and sensitivity analyses were carried out to explore the potential sources of heterogeneity caused by non-threshold effect when necessary [18]. The presence of publication bias in our included studies was evaluated by performing Deek’s funnel plot [19]. All analyses were conducted by using the Stata software (version 14.0; StataCorp.).

### Predicting targets of miR-29

The target genes of miR-29a and miR-29b were collected based on the powerful tool miRTarBase, which is a comprehensively annotated, experimentally validated and most recent updated miRNA-target interactions database in the field of miRNA associated research [20].

### Integrative functional annotation of of miR-29

To characterize the biomarker functions of miR-29 family (miR-29a and miR-29b) and further explore the mechanisms underlying the initiation and progression of CRC, we conducted GO and KEGG pathway enrichment analysis by employing the online tool Database for Annotation, Visualization and Integrated Discovery (DAVID) [21–23]. In the GO analysis, the categories include cellular component (CC), biological process (BP) and molecular function (MF) terms. The enriched GO terms and pathways with a P < 0.05 and gene count ≥ 2 were considered significant.

### PPI network analysis of miR-29 targets

The protein-protein interaction (PPI) information among the target genes of miR-29 family (miR-29a and miR-29b) were retrieved by uploading all the targets of miR-29a and miR-29b to the Search Tool for the Retrieval of Interacting Genes (STRING) database [24]. Based on the STRING database, PPIs of miR-29a and miR-29b targets were selected with score (median confidence) > 0.4, and PPI network was then visualized with the powerful tool Cytoscape [25]. In order to assess the importance of nodes in the PPI network, three common indices based on the plug-in CytoNCA, including betweenness centrality, closeness centrality and degree centrality, were investigated [26]. In addition, module analysis was employed to identify the significant modules within the constructed network by using the plug-in Molecular Complex Detection (MCODE) of Cytoscape. Moreover, DAVID was also utilized to perform function and pathway enrichment analysis for the identified hub nodes and the genes involved in the identified modules. P < 0.05 was considered to indicate a statistically significant difference.

## Results

### Study identification and characteristics of included studies

**Fig. 1:**
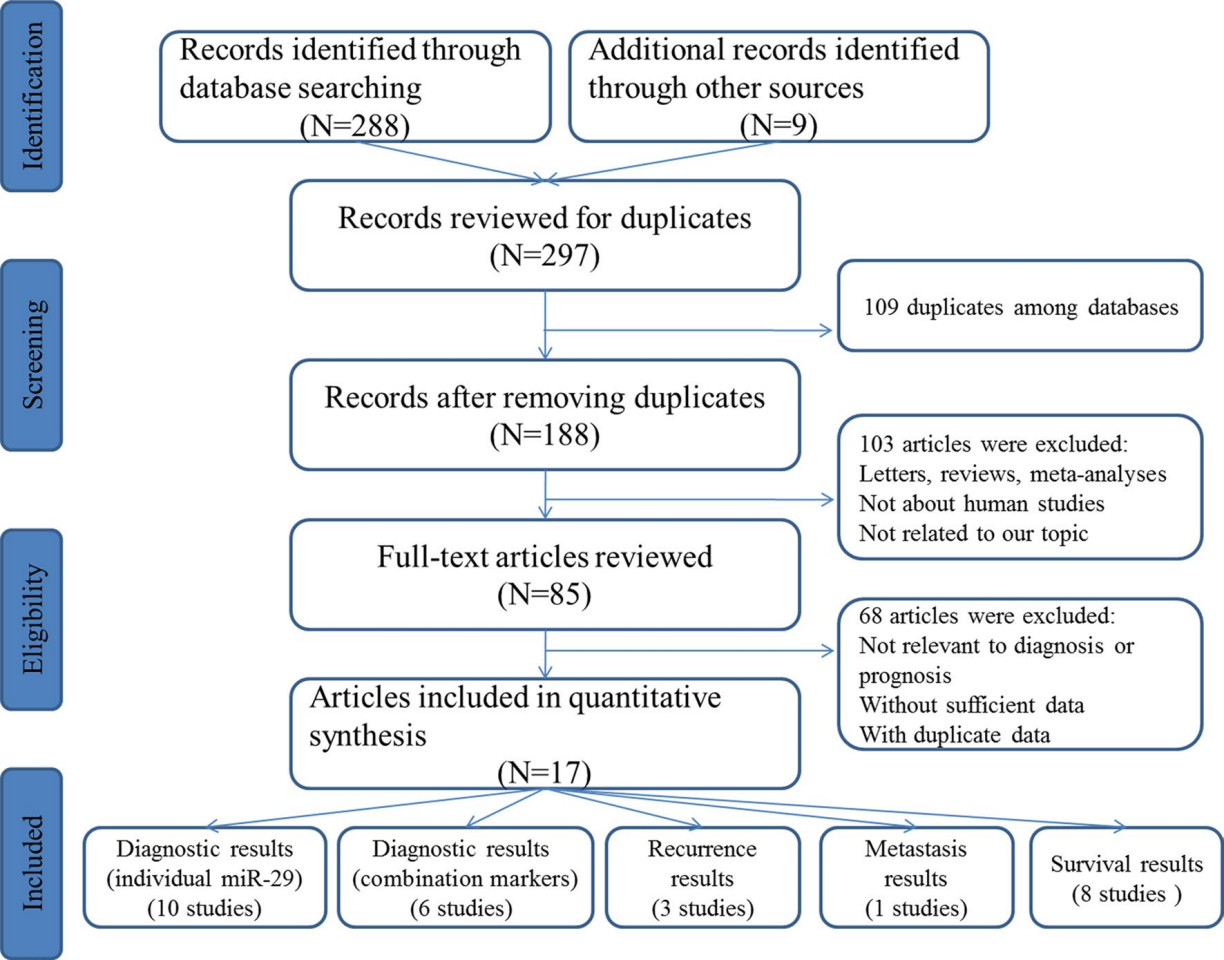
Flow diagram of the study selection process

The flowchart of detailed searching process was plotted in Fig. 1. Using the described searching strategy above, a total of 297 articles were initially retrieved out of the selected databases. After manually screening the titles, abstracts and keywords and then checking the full texts, 9 articles involving 10 studies for individual miR-29 and 5 articles containing 6 studies for miRNA combination markers based on miR-29 that met the inclusion norm were finally selected for the diagnostic meta-analysis while six publications including eight studies evaluating the survival outcome were identified eligible in the prognostic meta-analysis [27–39]. Moreover, three studies assessed the roles of miR-29 and miRNA combination markers in the recurrence prediction of CRC and one study evaluated the biomarker role in the metastasis prediction in CRC [40–43]. The characteristics of the eligible studies were summarized in Tables 1, 2 and 3. All these studies assessed miR-29 and miRNA combination biomarkers based on miR-29 expression by quantitative real-time PCR (qRT-PCR). The quality assessment results of all the eligible studies turned out to be from moderate to high.

**Table 1:** The main features of the included studies on individual miR-29 in the diagnosis of CRC

First author	Year	Country	Ethnicity	Case				Control				Sample source	Methods	miRNA	AUC	Sensitivity	Specificity	QUADAS
				M	F	N	Age	M	F	N	Age							
Huang [27]	2010	China	Asian	51	49	100	61	31	28	59	58	Plasma	RT-PCR	miR-29a	0.844	0.69	0.89	3
Giraldez [28]	2013	Spain	Caucasian	NA	NA	21	72.5	11	9	20	60.6	Plasma	RT-PCR	miR-29a	NA	0.62	0.85	4
Luo [29]	2013	Germany	Caucasian	45	35	80	68	60	84	144	62.5	Plasma	RT-PCR	miR-29a	0.571	0.30	0.90	4
Ramzy [34]	2015	Egypt	African	NA	NA	25	46	NA	NA	10	NA	Serum	RT-PCR	miR-29a	0.614	0.87	0.33	3
Basati [30]	2015	Iran	Asian	30	25	55	58.52	31	24	55	57.9	Serum	RT-PCR	miR-29b	0.87	0.77	0.75	4
Li [31]	2015	China	Asian	135	65	200	66.3	283	117	400	65.5	Tissue	RT-PCR	miR-29b	0.883	0.82	0.85	4
Li [31]	2015	China	Asian	135	65	200	66.3	283	117	400	65.5	Plasma	RT-PCR	miR-29b	0.743	0.61	0.72	4
Yamada [35]	2015	Japan	Asian	93	43	136	68	23	29	52	58	Serum	RT-PCR	miR-29a	0.741	0.60	0.84	3
Zhu [32]	2016	China	Asian	42	38	80	60.7	27	24	51	49.3	Feces	RT-PCR	miR-29a	0.777	0.85	0.61	4
Liu [33]	2018	China	Asian	51	34	85	59.5	48	30	78	34.8	Serum	RT-PCR	miR-29a	0.878	0.69	0.95	5

*M* male, *F* female, *N* number, *NA* not available, *AUC* area under the curve, *QUADAS* quality assessment of diagnostic accuracy studies

**Table 2:** The main features of the included studies on miR-29-related combination markers in the diagnosis of CRC

First author	Year	Country	Ethnicity	Case				Control				miRNA combinations	Sample source	Methods	AUC	Sensitivity	Specificity	QUADAS
				M	F	N	Age	M	F	N	Age							
Huang [27]	2010	China	Asian	51	49	100	61	31	28	59	58	miR-29a, miR-92a	Plasma	RT-PCR	0.883	0.83	0.85	3
Wang [36]	2012	China	Asian	NA	NA	90	NA	NA	NA	58	NA	miR-29a, miR-92a, miR-760	Plasma	RT-PCR	0.943	0.83	0.93	5
Luo [29]	2013	Germany	Caucasian	45	35	80	68	60	84	144	62.5	miR-29a, miR-92a, miR-18a, miR-20a, miR-21, miR-106b, miR-133a, miR-143, miR-145, miR-342-3p, miR-532-3p, miR-181b	Plasma	RT-PCR	0.745	0.72	0.75	4
Yamada [35]	2015	Japan	Asian	93	43	136	68	23	29	52	58	miR-29a, miR-21, miR-125b	Serum	RT-PCR	0.826	0.74	0.85	3
Liu [33]	2018	China	Asian	51	34	85	59.5	48	30	78	34.8	miR-29a, miR-92a	Serum	RT-PCR	0.876	0.73	0.94	5
Liu [33]	2018	China	Asian	51	34	85	59.5	48	30	78	34.8	miR-29a, miR-92a, miR-21, miR-125b	Serum	RT-PCR	0.952	0.85	0.99	5

*M* male, *F* female, *N* number, *NA* not available, *AUC* area under the curve, *QUADAS* quality assessment of diagnostic accuracy studies

**Table 3:** The main features of the included studies on miR-29 in the prognosis of CRC

First author	Year	Country	Ethnicity	M/F	N	Age	TNM stage	Sample source	miRNA	Methods	Endpoints	Follow-up time (months)	Hazard ratio	Scores
Brenner [42]	2011	Israel	Asian	34/25	59	78	II	Tissue	miR-29a	RT-PCR	DFS	NA	0.194 (0.063–0.597)	7
Tang [37]	2014	China	Asian	46/39	85	60	I-IV	Tissue	miR-29a	RT-PCR	OS	40	1.556 (1.066–2.282)	9
Inoue [38]	2014	Japan	Asian	153/92	245	NA	I-IV	Tissue	miR-29b	RT-PCR	OS	54	0.459 (0.240–0.850)	8
Inoue [38]	2014	Japan	Asian	153/92	245	NA	I-IV	Tissue	miR-29b	RT-PCR	DFS	54	0.452 (0.222–0.915)	7
Basati [30]	2015	Iran	Asian	30/25	55	58.5	I-IV	Serum	miR-29b	RT-PCR	OS	40	0.142 (0.029–0.342)	7
Yuan [40]	2017	China	Asian	81/63	144	56.1	I-IV	Plasma	miR-29a	RT-PCR	RFS	NA	2.610 (1.340–5.070)	7
Ulivi [39]	2018	Italy	Caucasian	35/17	52	65	I-IV	Plasma	miR-29b	RT-PCR	OS	NA	0.872 (0.753–0.991)	8
Ulivi [39]	2018	Italy	Caucasian	35/17	52	65	I-IV	Plasma	miR-29b	RT-PCR	PFS	NA	0.854 (0.728–0.997)	8

*M* male, *F* female, *N* number, *NA* not available, *DFS* disease-free survival, *OS* overall survival, *RFS* relapse-free survival, *PFS* progression-free survival

### Diagnostic value of individual miR-29 in detecting CRC

Among the included studies that evaluated individual miR-29 in detecting CRC, a total of 10 studies were identified enrolling 982 cases and 1269 healthy people as the control group. The sample types contained serum (n = 4), plasma (n = 4), feces (n = 1) and Tissue (n = 1). Overall, 7 studies were conducted in Asian populations, 2 in Caucasian populations and 1 in African populations.

**Fig. 2:**
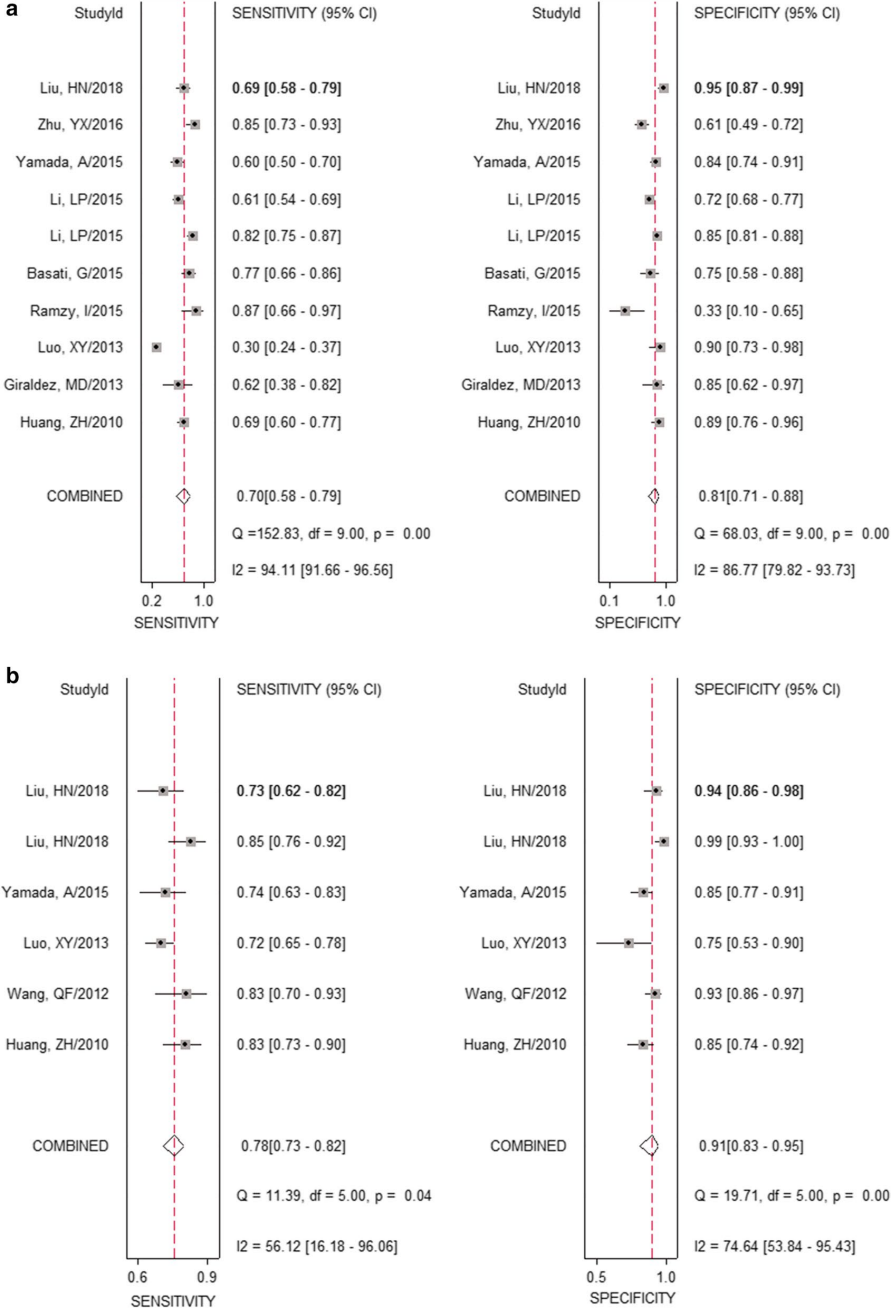
Forest plots of sensitivities and specificities from test accuracy studies in the diagnosis of CRC. **a** Forest plots of sensitivities and specificities for individual miR-29; **b** forest plots of sensitivities and specificities for combination markers based on miR-29

**Fig. 3:**
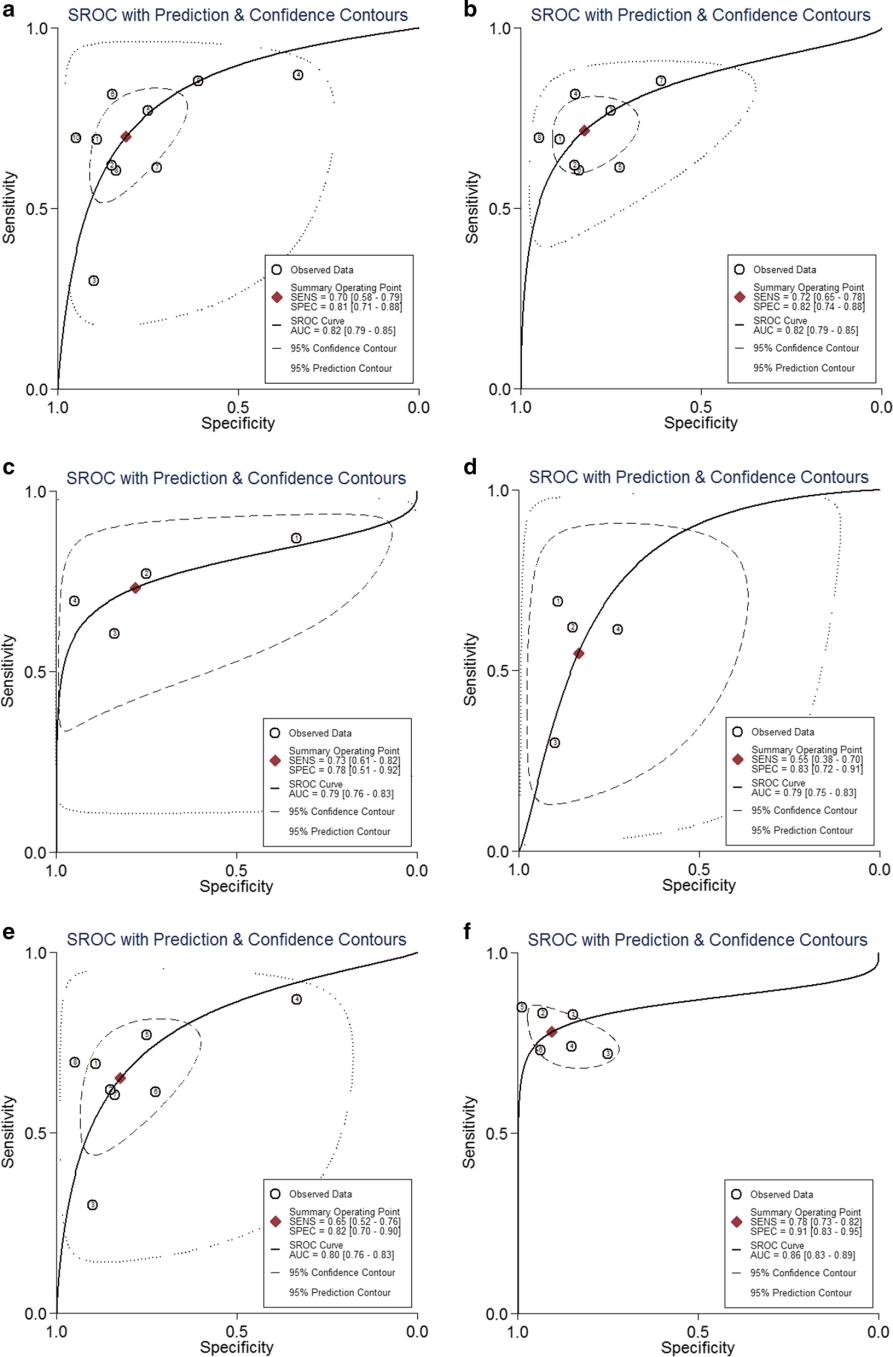
The SROC curves in the diagnosis of CRC. **a** SROC curve overall including the outliers for miR-29; **b** SROC curve of outliers excluded for miR-29; **c** SROC curve for miR-29 in serum samples; **d** SROC curve for miR-29 in plasma samples; **e** SROC curve for miR-29 in circulating samples; **f** SROC curve for combination markers based on miR-29. *SROC* summary receiver operator characteristic, *CRC* colorectal cancer

The forest plot of data from 10 studies about sensitivity and specificity is shown in Fig. 2. I^2^ values for sensitivity and specificity were 94.11 and 86.77%, respectively, while Q value for sensitivity and specificity were 152.83 (P < 0.01) and 88.03 (P < 0.01), respectively, indicating that statistical heterogeneity existed between studies. The overall pooled results for sensitivity, specificity, and DOR were 70% (95% CI 58–79%), 81% (71–88%) and 10 (6–17) respectively. Since the likelihood ratios (PLR and RLR) have been considered more clinically valuable than specificity and sensitivity. In detail, PLR > 10 or NLR < 0.1 indicates high diagnostic accuracy. In the present study, the pooled PLR is 3.7 (2.5–5.5), indicating that the CRC patients have nearly a four–fold probability of being miR-29 positive in comparison to healthy individuals. The pooled NLR was 0.37 (0.27–0.51), which suggested that expected proportion of patients suffering from CRC is 37% if the miR-29 is negative. The SROC curve (Fig. 3) was generated and the AUC was 0.82 (0.79–0.85), suggesting miR-29 has a moderate diagnostic power in CRC.

To exclude the heterogeneity from the threshold effect, a Spearman rank correlation was conducted between the logit of sensitivity and logit of 1-specificity. According to the result, the absence of heterogeneity was validated from the threshold effect since the Spearman correlation coefficient was − 0.65 with the P value of 0.42 (P > 0.05).

In order to identify potential sources of inter-study heterogeneity in the overall estimates, we performed subgroup analyses (Table 4). There is no big difference between the pooled data in serum-based and plasma-based miR-29 assays, with a sensitivity of 0.73 versus 0.55, specificity of 0.78 versus 0.83, PLR of 3.3 versus 3.3, NLR of 0.34 versus 0.54, DOR of 10 versus 6 and AUC of 0.79 versus 0.79 (Fig. 3). However, since the pooled PLR, NLR and DOR have been considered as more valuable parameters than sensitivity or specificity for clinical applications, we considered serum may be a better matrix for screening CRC in miRNA method. Among the ten studies, eight detected the miRNA assay in circulating samples. Therefore, subgroup analysis was also carried out by circulating samples (Fig. 3). In total, the pooled sensitivity, specificity, and AUC of circulating miR-29 were 0.65 (0.52–0.76), 0.82 (0.70–0.90), and 0.80 (0.76–0.83), respectively. In addition, the diagnostic sensitivity, specificity, PLR, NLR, and DOR of Asian-based miRNA assays were 0.72 (0.65–0.79), 0.82 (0.73–0.89), 4.0 (2.6–6.2), 0.34 (0.26–0.43), and 12 (7–21), respectively, with AUC of 0.83 (0.79–0.86). We also found that pooled studies with large sample size assays showed a higher level of overall accuracy compared with that of small sample size, with a sensitivity of 0.61 versus 0.78, specificity of 0.86 versus 0.73, PLR of 4.3 versus 2.9, NLR of 0.45 versus 0.30, DOR of 10 versus 10, and AUC of 0.84 versus 0.82, which indicated that more large-scale prospective studies are warranted.

**Table 4:** Pooled results of diagnostic accuracy of miR-29 and combination biomarkers in CRC

	Analysis	Number of studies	Se (95%CI)	Sp (95%CI)	AUC (95%CI)
Individual	Ethnicity				
	Asian	7	0.72 (0.65–0.79)	0.82 (0.73–0.89)	0.83 (0.79–0.86)
	Sample type				
	Plasma	4	0.55 (0.38–0.70)	0.83 (0.72–0.91)	0.79 (0.75–0.83)
	Serum	4	0.73 (0.61–0.82)	0.78 (0.51–0.92)	0.79 (0.76–0.83)
	Circulating	8	0.65 (0.52–0.76)	0.82 (0.70–0.90)	0.80 (0.76–0.83)
	Feces	1	0.85 (0.73–0.93)	0.61 (0.49–0.71)	0.78 (0.69–0.86)
	Tissue	1	0.82 (0.75–0.87)	0.85 (0.81–0.88)	0.88
	Sample size				
	> Median	5	0.61 (0.45–0.76)	0.86 (0.77–0.92)	0.84 (0.81–0.87)
	< Median	5	0.78 (0.68–0.85)	0.73 (0.54–0.86)	0.82 (0.79–0.85)
	miRNA profiling				
	miR-29a	7	0.68 (0.52–0.81)	0.83 (0.67–0.92)	0.82 (0.79–0.85)
	miR-29b	3	0.72 (0.68–0.77)	0.77 (0.76–0.81)	0.86 (0.78–0.94)
	Overall	10	0.70 (0.58–0.79)	0.81 (0.71–0.88)	0.82 (0.79–0.85)
	Outliers excluded	8	0.72 (0.65–0.78)	0.82 (0.74–0.88)	0.82 (0.79–0.85)
Combination	Overall	6	0.78 (0.73–0.82)	0.91 (0.83–0.95)	0.86 (0.83–0.89)

*AUC* area under the curve, *Se* sensitivity, *Sp* specificity, *95% CI* 95% confidence interval

Then, we attempted to further explain the heterogeneity by conducting the meta-regression analysis. We considered 4 covariates (ethnicity, sample size, sample source and miR-29 type) may contribute to the heterogeneity. The results revealed that neither ethnicity, nor sample size or miR-29 type was the source of heterogeneity, but the sample source may result in the heterogeneity (P < 0.05).

**Fig. 4:**
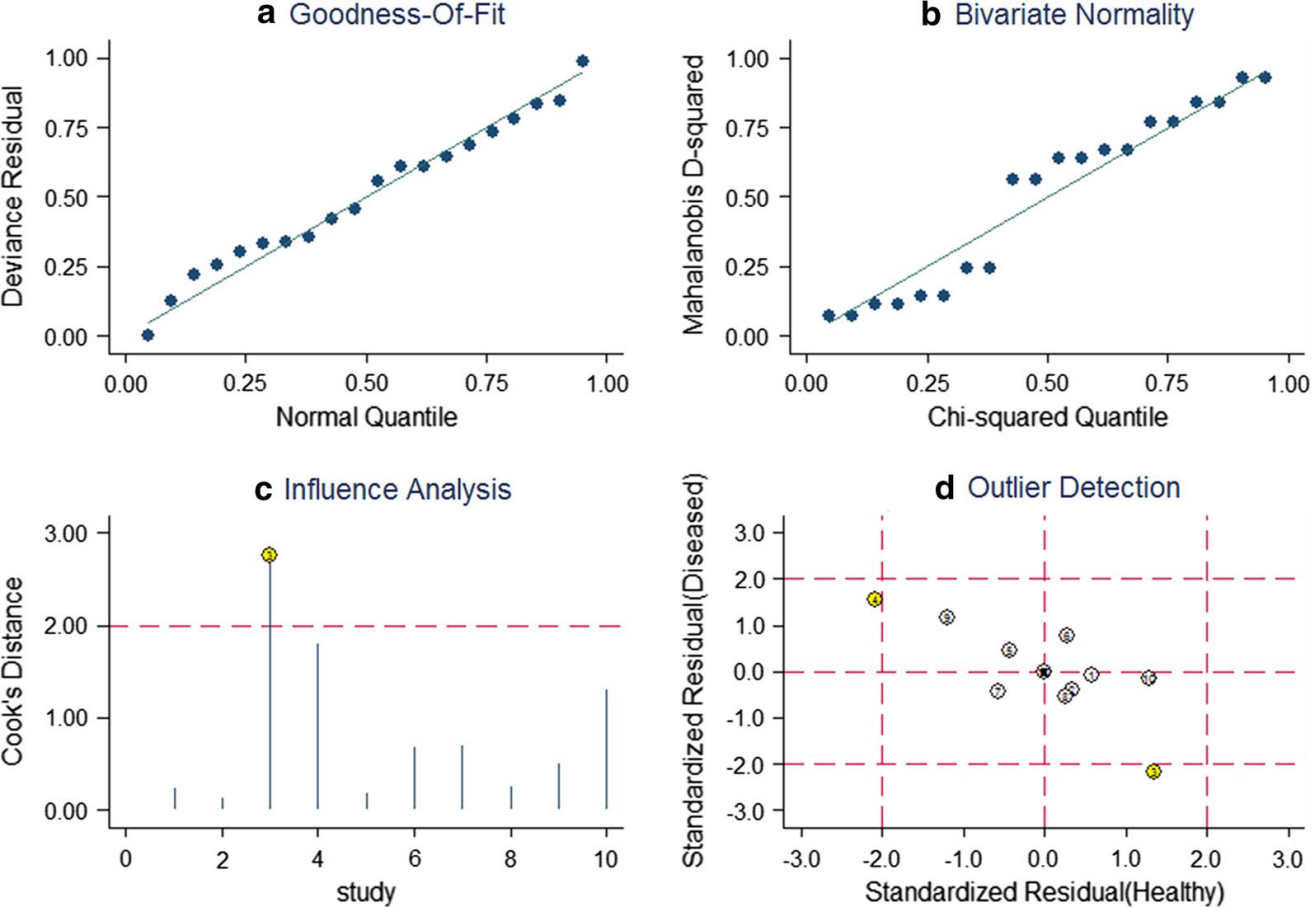
Sensitivity analysis results for individual miR-29 in the diagnosis of CRC. **a** Goodness of fit; **b** bivariate normality; **c** influence analysis; **d** outlier detection

Sensitivity analyses were further carried out to investigate the effect of single study on the overall conclusion. Goodness of fit and bivariate normality analyses (Fig. 4) revealed that the bivariate random-effects model was robust for the evidence synthesis of individual miR-29. There were two deviated studies that may overshadow the diagnostic power of miR-29 according to the influence analysis and outlier detection (Fig. 4). After excluding the deviated studies, the I^2^ of sensitivity decreased from 94.11 to 76.45% and that of specificity increased from 86.77 to 87.07%. However, there were only minimal changes in the pooled estimates of sensitivity (0.70 vs. 0.72), specificity (0.81 vs. 0.82), PLR (3.7 vs. 4.0), NLR (0.37 vs. 0.35), DOR (10 vs. 12), and AUC (0.82 vs. 0.82) between the overall analysis with and without outliers (Fig. 3), indicating that our meta-analysis was highly robust.

**Fig. 5:**
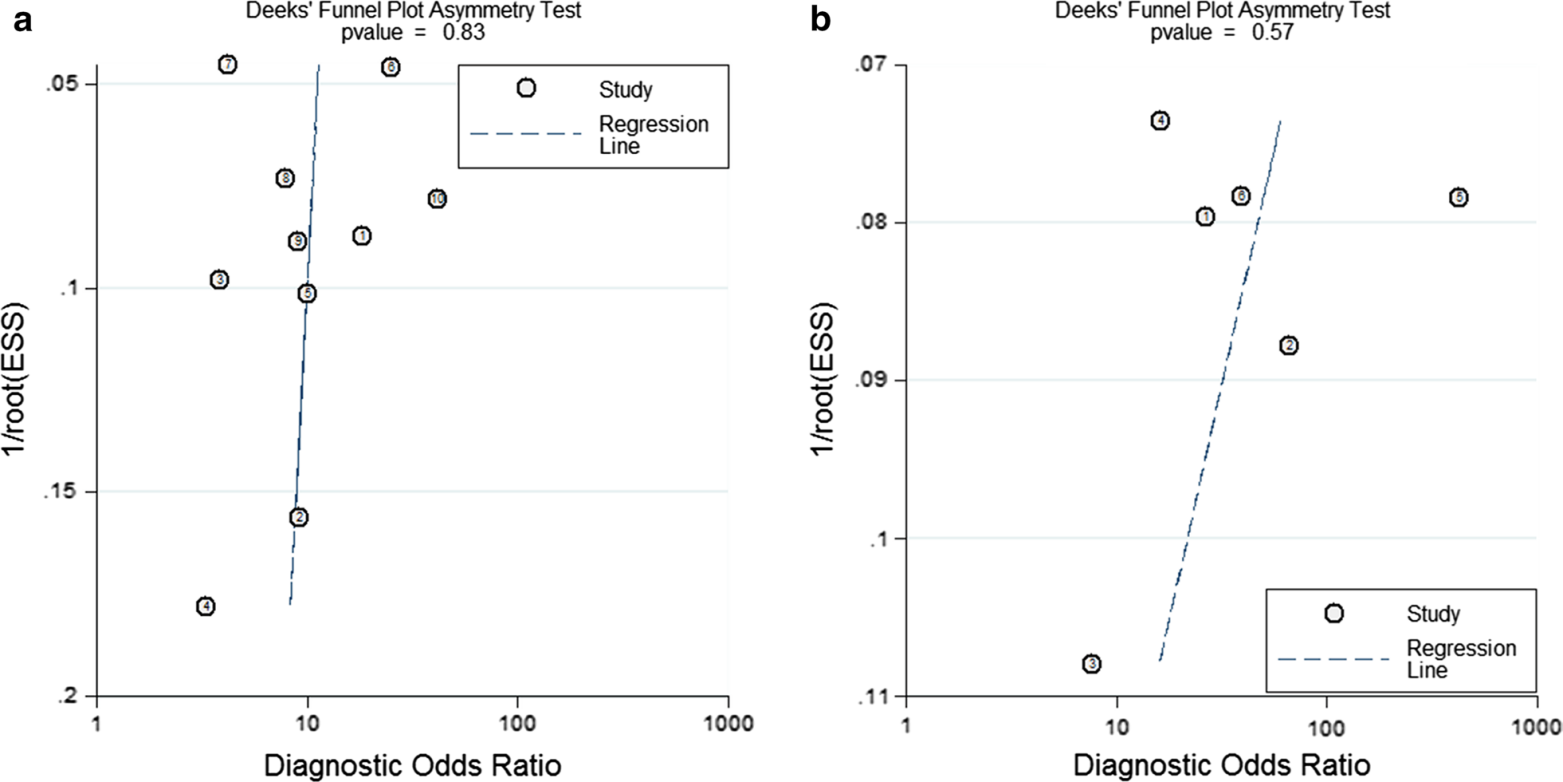
Funnel plots for the assessment of potential bias in the meta-analysis for diagnosis. **a** Funnel plot of the studies on individual miR-29; **b** funnel plot of the studies on combination markers based on miR-29

Finally, potential publication bias was evaluated using funnel plots. As a result, no significant publication bias was observed in this meta-analysis for assessing the biomarker role of individual miR-29 (P = 0.83) (Fig. 5).

### Diagnostic value of miRNA combination markers based on miR-29 in detecting CRC

A total of 6 studies involving 491 cancer patients and 391 healthy people were included in the analysis for miRNA combination markers based on miR-29. Among them, 3 were plasma and 3 were serum samples.

There is also significant heterogeneity among the sensitivity and specificity results in the studies evaluating miRNA combination markers based on miR-29. For the sensitivity, the I^2^ was 56.12% (95% CI 16.18–96.06), and Q value was 11.39 (P = 0.04); for the specificity, the I^2^ was 74.64 (53.84–95.43), and Q value was 19.71 (P < 0.01). Overall, the pooled assessment outcomes were as follows: sensitivity, 0.78 (95% CI 0.73–0.82); specificity, 0.91 (0.83–0.95); PLR, 8.3 (4.3–16.1); NLR, 0.24 (0.19–0.31); and DOR, 34 (14–81), respectively (Fig. 2). The area under the summary ROC curve is presented at Fig. 3 with AUC of 0.86 (95% CI 0.83–0.89), indicating a relatively higher accuracy. We further compared them with individual miR-29 and the results revealed that miRNA combination biomarkers based on miR-29 had a higher level of predictive power than individual miR-29, with sensitivity of 0.78 (0.73–0.82) vs. 0.70 (0.58–0.79), specificity of 0.91 (0.83–0.95) vs. 0.81 (0.71–0.88), and AUC of 0.86 (0.83–0.89) vs. 0.82 (0.79–0.85).

The Spearman correlation coefficient revealed that there was no heterogeneity generating from threshold effect (P > 0.05). Goodness of fit and bivariate normality analyses confirmed that the selected analysis model was robust for the calculation of the pooled estimate. No outlier study was identified from the influence analysis and outlier detection. The shape of the funnel plot indicates evidence of asymmetry (Fig. 5) with P value of 0.57, indicating that there was no significant publication bias in the analysis for evaluating miRNA combination biomarkers. The above tests suggest the robustness of our meta-analysis’s results for evaluating miRNA combination biomarkers. However, due to the limited number of studies, we could not perform further analysis including subgroup and meta-regression analyses.

### Recurrence prediction value of miR-29 and the combination markers in CRC

One study evaluated the diagnostic role of miR-29a and the related combination markers in the recurrence of CRC. In the study, miR-29a could discriminate recurred patients from non-recurred stage III CRC patients with a sensitivity of 50.0%, a specificity of 42.9% and the AUC of 0.452 while the combination markers (miR-29a, miR-92, miR-17 and miR-21) increased the diagnostic power for the patients, yielding an AUC of 0.881, with a sensitivity of 83.3% and a specificity of 85.7% (P < 0.05) [41]. In another study, the sensitivity of miR-29a for identifying recurrence in CRC was 31.0%, the specificity was 98.0% [42]. In the third study, miR-29a showed promising prognostic significance and could discriminate patients with recurrence from those without recurrence with an AUC of 0.703 (95% CI 0.562, 0.845). Meanwhile, in this study, preoperative high plasma miR-29a levels were associated with increased recurrence risk (HR 2.61; 95% CI 1.34–5.07; P < 0.005) [40].

### Metastasis prediction value of miR-29 in CRC

There was one study that evaluated the value of miR-29 for predicting the metastasis in CRC [43]. In the study, serum miR-29a was found significantly higher in colorectal liver metastasis patients than in CRC patients, yielding a AUC of 80.3% with the sensitivity of 75% and the specificity of 75% in discriminating metastatic from non-metastatic patients, revealing that serum miR-29a may have strong potential to be a promising noninvasive biomarker for early detection of CRC with liver metastasis.

### Survival prediction value of miR-29 in CRC

A total 927 patients among the included studies were summarized to evaluate the impact of miR-29 expression on the survival outcome of CRC. Due to obvious heterogeneity (I^2^ = 85.9%; P < 0.001), random-effects model was applied to calculate pooled HR and its 95% CI. The results suggested that higher expression level of miR-29 family was associated with better survival outcome (OS/DFS/RFS/PFS) in patients with CRC (HR 0.78; 95% CI 0.56–1.07; P = 0.127). For the endpoint of OS, miR-29 family high expression was demonstrated to moderately predict better OS (HR = 0.69, 95% CI 0.39–1.25, P = 0.220). Subgroup analyses by miR-29 classification showed that elevated miR-29b yielded a better survival (OS/DFS/RFS/PFS) in CRC patients with combined HR of 0.68 (95% CI 0.51–0.90, P = 0.007) compared with miR-29a (HR = 1.04, 95% CI 0.36–3.07, P = 0.937). In addition, when analyzing the studies by the sample source, it was found that the pooled HR of survival outcome (OS/DFS/RFS/PFS) was 0.54 (95% CI 0.22–1.33, P = 0.183) in tissue sample and 0.92 (95% CI 0.64–1.31, P = 0.638) in circulation sample, respectively.

### Integrative functional analysis results of miR-29

**Fig. 6:**
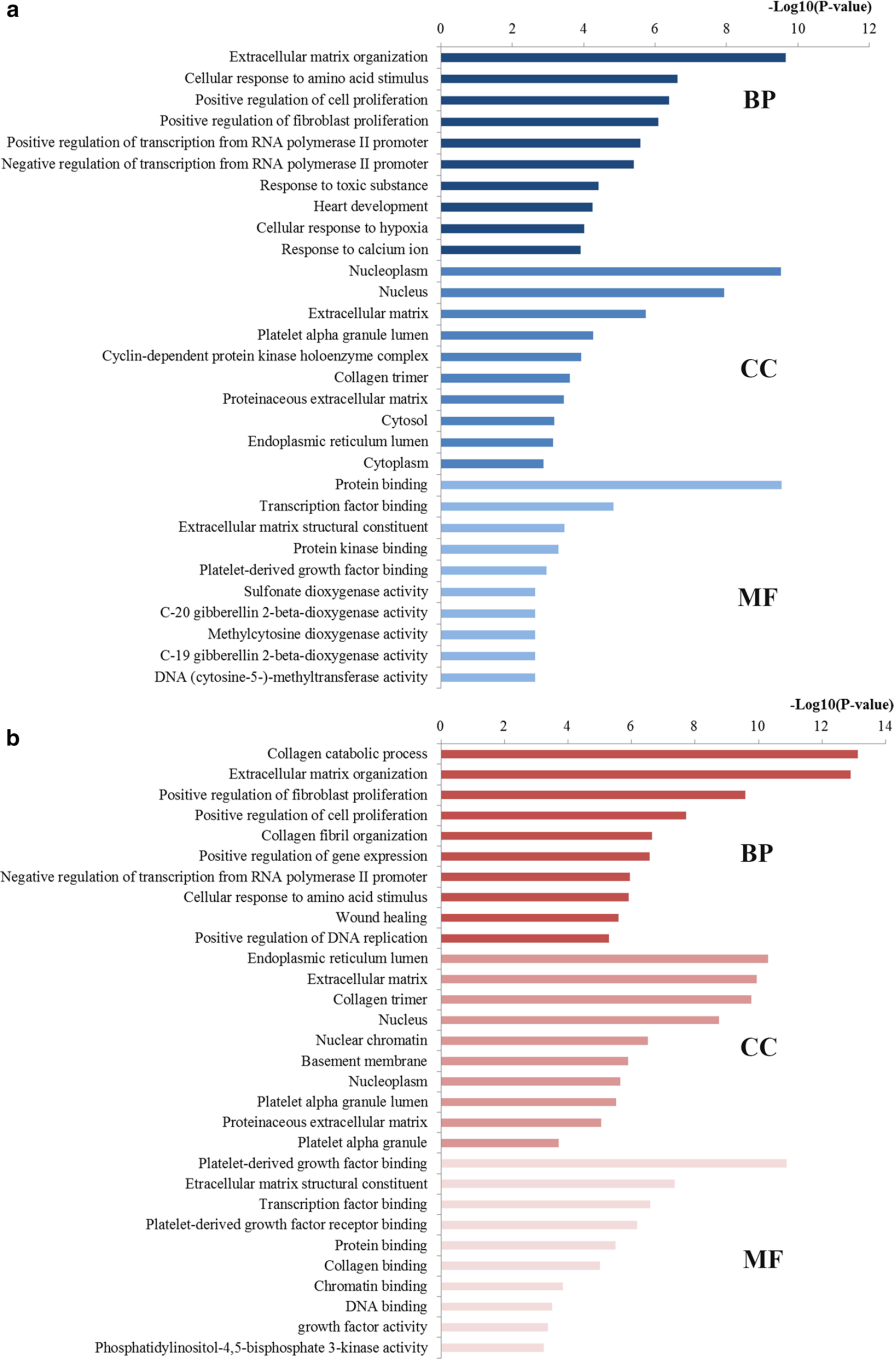
GO annotation of miR-29 target genes. **a** Top 10 GO items for target genes of miR-29a; **b** Top 10 GO items for target genes of miR-29b. *GO* gene ontology, *BP* biological processes, *CC* cell component, *MF* molecular function

To characterize the biological functions of miR-29 family, we obtained the GO functions and pathways through the target genes enrichment analysis of miR-29a and miR-29b. After conducting the significance analysis of GO terms for the target genes of miR-29a and miR-29b, we identified the top ten significant GO terms of each category. Figure 6 indicates that in the BP category for miR-29a, the targeted genes were mainly linked with the regulation processes including regulation of cell proliferation, fibroblast proliferation, transcription from RNA polymerase II promoter and response to amino acid stimulus, toxic substance and hypoxia. In the CC category for miR-29a, the target genes were closely relevant to some cellular components such as nucleoplasm, nucleus and extracellular matrix. In the MF category for miR-29a, the target genes were highly involved the aspects of protein binding, transcription factor binding and protein kinase binding. As also shown in Fig. 6, at the BP level for miR-29b, the most significant terms were mainly associated with positive regulation of fibroblast proliferation, positive regulation of cell proliferation and positive regulation of gene expression. At the CC level for miR-29b, the enriched terms were highly concentrated on nucleus, nuclear chromatin, basement membrane and nucleoplasm. At the MF level for miR-29b, most enriched terms were closely linked with platelet-derived growth factor binding, transcription factor binding, platelet-derived growth factor receptor binding and protein binding.

**Fig. 7:**
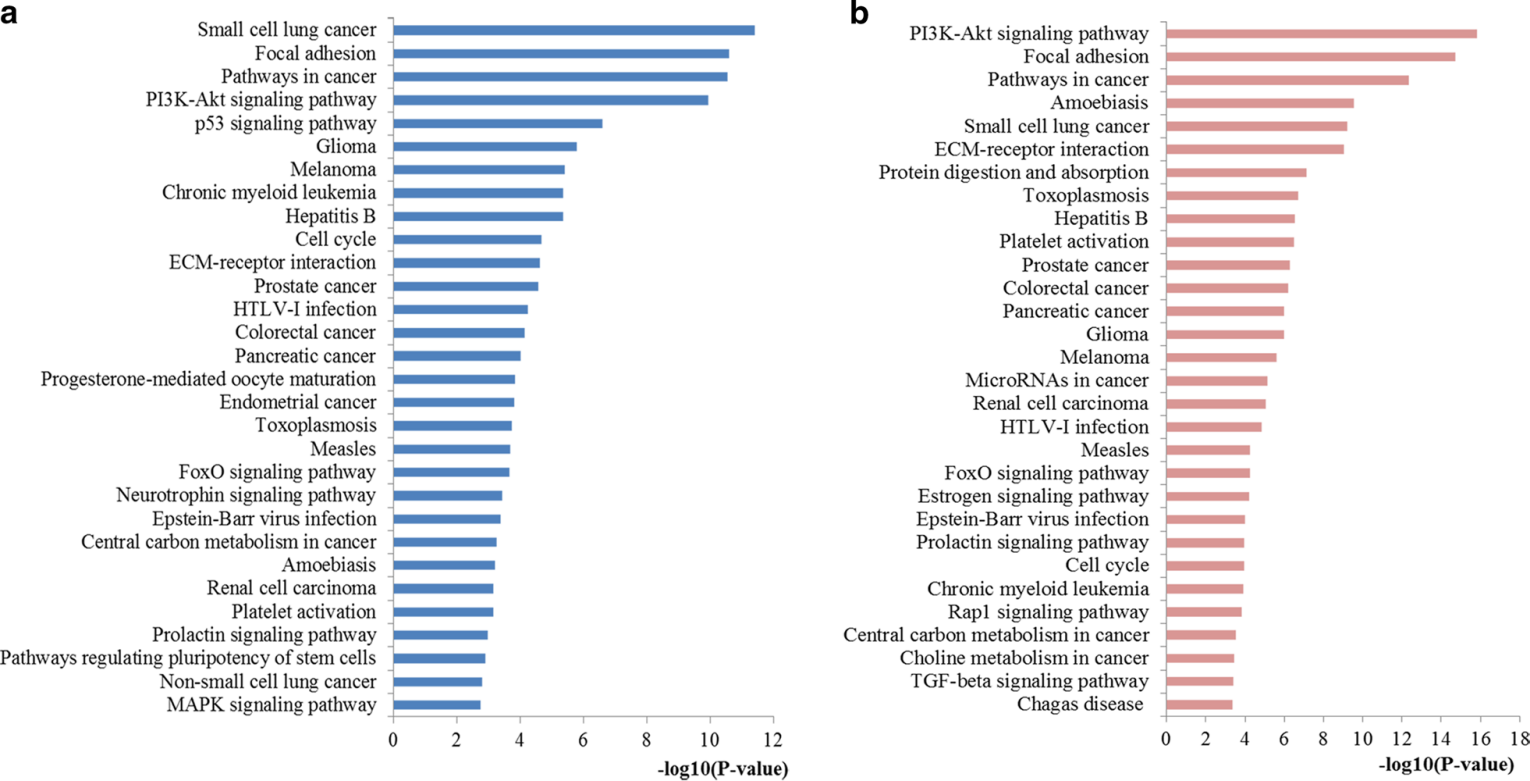
Pathway enrichment results for miR-29 target genes. **a** Top 30 pathways enriched by target genes of miR-29a; **b** top 30 pathways enriched by target genes of miR-29b

The pathway enrichment analysis of miR-29a and miR-29b was further conducted. Here we mainly focused on the top 30 significantly enriched terms for in-depth analyses (Fig. 7). It was revealed from the results that the target genes of miR-29a were mainly related to the pathways including focal adhesion, pathways in cancer, PI3K-Akt signaling pathway, p53 signaling pathway, cell cycle, colorectal cancer and FoxO signaling pathway. Meanwhile, the target genes of miR-29b were highly involved in the pathways such as PI3K-Akt signaling pathway, focal adhesion, pathways in cancer, colorectal cancer, microRNAs in cancer and FoxO signaling pathway.

By means of GO and pathway enrichment analysis of miR-29 targets, it can help us comprehend how the identified miRNA affect the biological pathways implicated in the development of CRC.

### PPI network construction and analysis of miR-29 targets

**Fig. 8:**
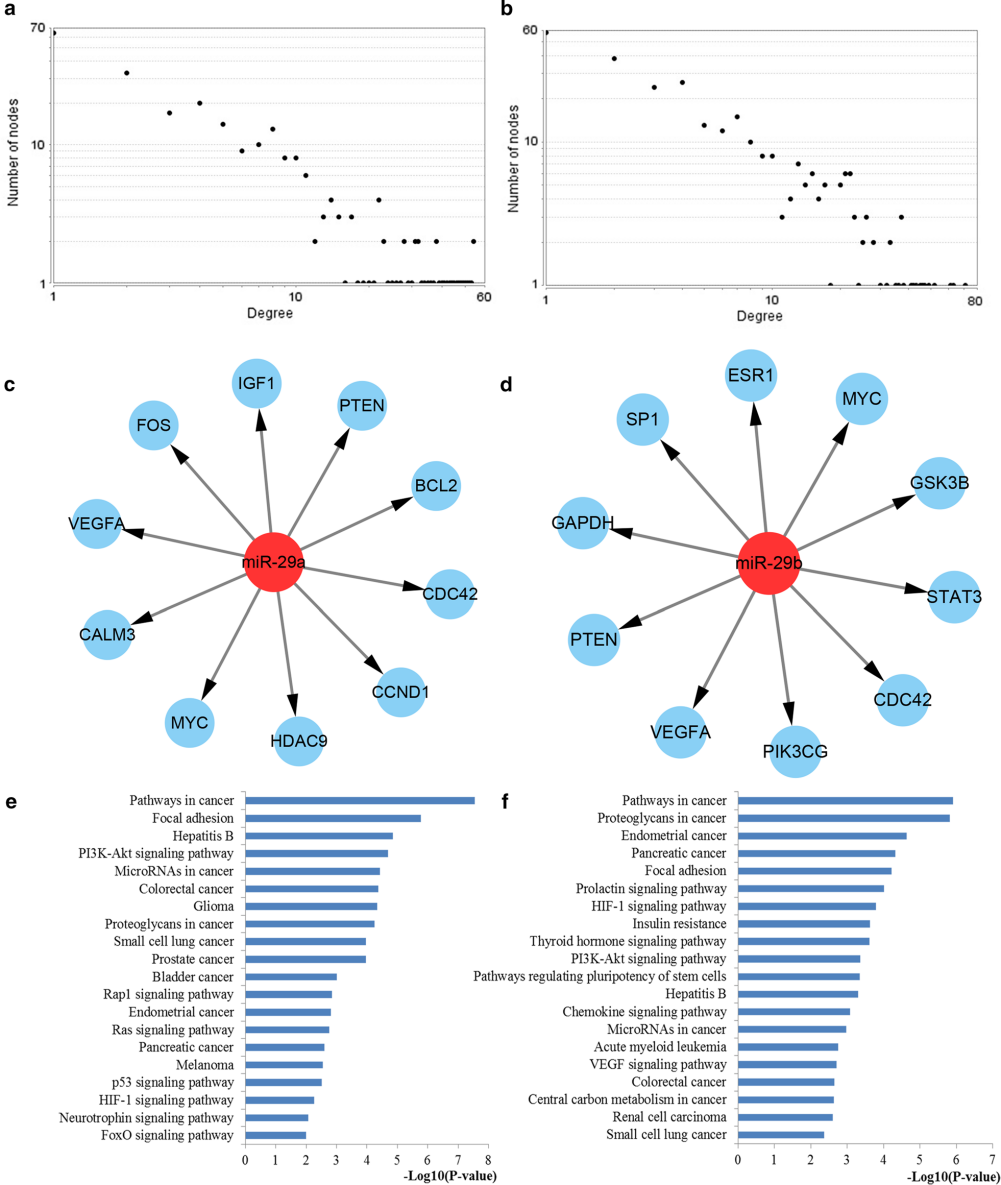
PPI network construction results. **a** Degree distributions of nodes for network constructed with miR-29a targets; **b** degree distributions of nodes for network set up with miR-29b targets; **c** hub genes of network for miR-29a targets; **d** hub genes of network for miR-29b targets; **e** pathway enrichment results for the selected hub genes of miR-29a targets network; **f** pathway enrichment results for the selected hub genes of miR-29b targets network. *PPI* protein–protein interaction

To obtain an improved understanding of the pathogenic mechanisms of miR-29 family in CRC occurrence and development, we further evaluated the internal contact and interactions among the target genes of miR-29a and miR-29b. After the identification of the PPI information among the target genes of miR-29a and miR-29b from the STRING database, the PPI networks were constructed and visualized made up of 248 and 296 nodes for miR-29a and miR-29b with statistical significance by using the Cytoscape platform software, respectively. The degree distributions of the network nodes were illustrated in Fig. 8. Then the crucial hub genes of the PPI networks for miR-29a and miR-29b targets were identified based on three different centrality measures. The top ten hub nodes regulated by miR-29a and miR-29b were also plotted in Fig. 8.

Functional enrichment analyses were performed to explore the function of these key hub nodes (Fig. 8). According to the enrichment results, the hub nodes of the network for miR-29a targets were significantly enriched into several important pathways including pathways in cancer, focal adhesion, PI3K-Akt signaling pathway, microRNAs in cancer, colorectal cancer and proteoglycans in cancer while the hub nodes of the network for miR-29b targets were linked with pathways in cancer, proteoglycans in cancer, HIF-1 signaling pathway, PI3K-Akt signaling pathway, microRNAs in cancer, colorectal cancer and VEGF signaling pathway. These pathways were proved associated with the establishment and development of CRC.

**Fig. 9:**
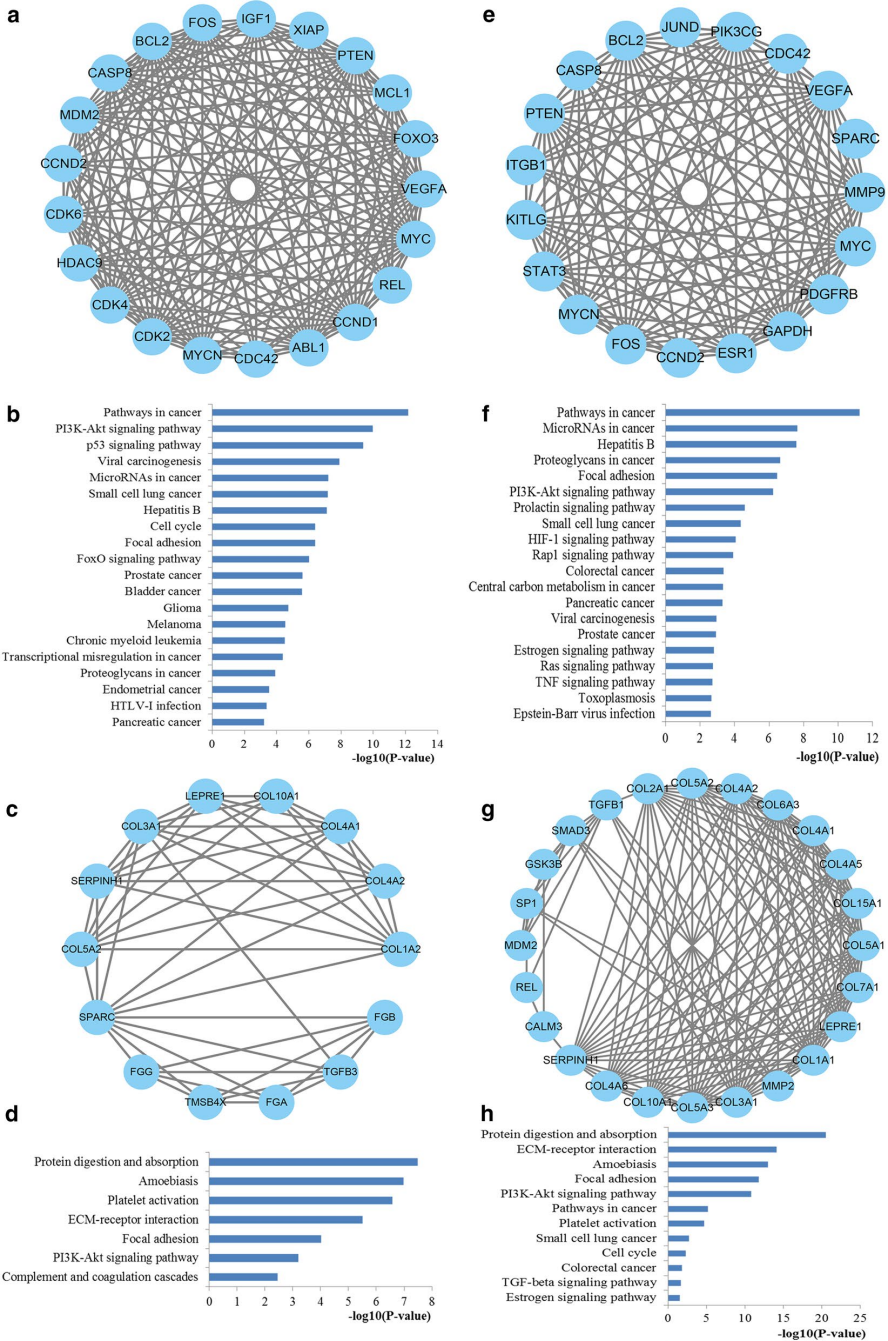
Module analysis results from the PPI network. **a**, **c** The significant modules in the PPI network for miR-29a targets; **b**, **d** Pathways enriched by all the nodes involved in the identified modules for miR-29a; **e**, **g** The significant modules in the PPI network for miR-29b targets; **f**, **h** Pathways enriched by all the nodes involved in the screened modules for miR-29b. PPI protein–protein interaction

Based on the MCODE package, the significant network modules were retrieved from the PPI network (Fig. 9). Then the genes involved in the identified modules were enriched by KEGG pathway analysis. As a result, the genes involved in the significant modules of miR-29a targets network were highly associated with a series of significant pathways such as pathways in cancer, PI3K-Akt signaling pathway, p53 signaling pathway, microRNAs in cancer, cell cycle and FoxO signaling pathway. Meanwhile, the genes associated with the identified modules of miR-29b targets network were mainly related to some important pathways including pathways in cancer, microRNAs in cancer, proteoglycans in cancer, PI3K-Akt signaling pathway, HIF-1 signaling pathway and colorectal cancer.

## Discussion

CRC, a fatal disease, has attracted increasing attention from clinicians across the world due to its high morbidity and mortality rates. Early stage diagnosis of the disease by noninvasive approaches could lead to effective treatment and better consequences. However, current diagnostic and prognostic methods for CRC were either invasive or expensive. Promisingly, the discovery of miRNAs has opened a window of a non-invasive test for the early detection and survival prediction of CRC. As promising miRNA candidates, the utility of miR-29 family members as considerable markers of CRC progression and diagnosis have previously been investigated. Nevertheless, different studies generated some conflicting results, which have prevented its application to clinical practice. Thus, we systematically reviewed the published studies and performed this comprehensive and up-to-date research to draw a complete overview of all reported clinical studies assessing the biomarker value of miR-29 family in the diagnosis and prognosis of CRC patients. Meanwhile, an integrated bioinformatics analysis was carried out to promote the understanding of underlying mechanisms of miR-29 family in the establishment and development of CRC.

In this study, we found that miR-29 achieved the overall pooled sensitivity of 0.70 (95% CI 0.58–0.79), specificity of 0.81 (0.71–0.88), and AUC of 0.82 (0.79–0.85). The results indicated its moderate diagnosis power of CRC as noninvasive detection. Compared with traditional biomarkers such as CEA and CA19-9 which have been widely used as tumor markers for CRC in clinical settings, our results also indicated that miR-29 has superior diagnostic properties for CRC screening due to its higher sensitivity, noninvasiveness, and simple detection. Meanwhile, it is worth noting that the sample sources exerted an impact on the diagnostic power as serum-based miR-29 assays achieved slightly higher overall diagnostic accuracy than plasma-based miR-29 assays according to the subgroup analysis. Moreover, consistent with the conclusion provided by the subgroup analysis, meta-regression analysis also revealed the sample sources were associated with the diagnostic power.

Nowadays, great efforts have been undertaken in search of cancer biomarkers. However, most attention concentrated on single or limited molecules. As known to all, CRC is a heterogeneous disease with a complex etiology. The occurrence and progression of CRC is commonly due to multistep, multifactor, and polygenic effects and involves changes in various oncogenes and tumor associated miRNAs. Therefore, combination biomarkers may be more qualified with higher prediction power than single markers. Therefore, a systematic evaluation of the published studies was carried out to investigate the diagnostic values of combination biomarkers based on miR-29 in CRC. Our results revealed that utilizing combination biomarkers based on miR-29 for the detection of CRC yielded an overall sensitivity of 78% and an overall specificity of 91% with the AUC of 0.86. Our results revealed that miRNA combination biomarkers based on miR-29 has a prominent advantage over single miR-29 for CRC screening because of its higher sensitivity and specificity.

The biggest challenge of CRC treatment is local recurrence and distant metastasis. Exploring sensitive and specific biomarkers is also in urgent need for early prediction of local recurrence and distant metastasis. Promisingly, according to our results, miR-29 family may also serve as novel biomarkers for predicting the recurrence and metastasis of CRC. It must be noted that, miRNA combination biomarkers based on miR-29 further improved the prediction accuracy of local recurrence.

Previously, miR-29 family was reported to act as tumor suppressors or oncogenes in diverse cancers. Recent studies investigating its association with CRC outcomes have disclosed the prognostic value of miR-29 family. However, consensus has not been reached as to the reliability of miR-29 family as prognostic markers in CRC due to some opposite results. For the prognosis evaluation, our result indicated that miR-29 expression level is a potential biomarker for predicting survival outcomes in CRC patients. It was revealed from results that high miR-29 may be related to better survival with the pooled HR of 0.78 (0.56–1.08) although the result has not reach the statistical significance. However, it came to more obvious with statistical significance for miR-29b that over-expression of miR-29b in CRC was predictive of better outcome. Of course, the prognostic value for miR-29 family still remains controversial due to limited number of studies enrolled in our analysis and more clinical studies are warranted.

The biological function of miR-29 family may affect the relationship between miR-29 family expression and CRC. Therefore, we further investigated the pathogenic mechanisms of the miR-29 family in CRC occurrence and development through an integrated bioinformatics analysis. GO enrichment analysis is a functional method designed for annotating large numbers of genes. According to the enrichment analysis, most GO terms enriched by the target genes of miR-29a and miR-29b both mainly focused on the processes of regulation at the BP level, significantly relevant to core cell structural at CC level and highly linked with the function of binding at the MF level, which convinced the underlying biomarker power of miR-29 family for CRC initiation and progression predicting. Pathway enrichment analysis may reveal more precise information regarding biological functions compared with GO analysis. The results indicated that the genes targeted by miR-29a and miR-29b were enriched into similar pathways strongly associated with CRC initiation and development, such as pathways in cancer, PI3K-Akt signaling pathway, p53 signaling pathway, cell cycle, colorectal cancer and FoxO signaling pathway and microRNAs in cancer. For example, the pathways in cancer, colorectal cancer and microRNAs in cancer signaling pathways directly reflect the associations among miR-29a and miR-29b target genes and establishment and progression of CRC. Accumulating new evidence has indicated that PI3K-Akt signaling pathway leads to reduced apoptosis, stimulates cell growth and increases proliferation [44]. Aberrant activation of PI3K-Akt signaling has been convinced as a critical event in the development of CRC [45]. The well-studied p53 pathway, one of the most important pathways in carcinogenesis, plays a central part in cell-intrinsic responses to genome instability, including a transient cell cycle arrest, senescence and apoptosis [46]. It is well established that p53 signaling is involved in the establishment and progression of almost all types of cancer including CRC [47]. The cell cycle pathway, another very important signaling pathway, has been critically reviewed by a large amount of studies for its pathogenesis in malignant progression of a variety of human cancers including CRC due to its multifunctional roles in cell growth, inflammation, differentiation, apoptosis, and metastasis [48]. Recent new evidence gathered so far has indicated that FoxO signaling pathway play not solely tumor suppression roles, but also support tumor growth and metastasis by regulating a multitude of cellular processes essential for tumorigenesis [49]. The functional enrichment analysis characterized the biomarker properties of miR-29 family, contributed to the function and molecular mechanism of miR-29 family in CRC establishment and progression and implied that miR-29 family might be employed as promising biomarkers for CRC.

Since close associations exist among the target genes of miR-29a and miR-29b, PPI network analysis was conducted to further explore the relationships among miR-29a and miR-29b targets. Through PPI network construction, several key hub genes regulated by miR-29a and miR-29b were identified and both enriched into a series of CRC related signaling pathways including pathways in cancer, PI3K-Akt signaling pathway, microRNAs in cancer, colorectal cancer and proteoglycans in cancer, HIF-1 signaling pathway, and VEGF signaling pathway. Furthermore, module analysis of the PPI network identified several significant modules. It was indicated from the functional enrichment results that the genes involved in the screened modules of the networks for miR-29a and miR-29b both played important roles in several signaling pathways associated with the occurrence and development of CRC such as pathways in cancer, PI3K-Akt signaling pathway, p53 signaling pathway, microRNAs in cancer, cell cycle, FoxO signaling pathway, proteoglycans in cancer, HIF-1 signaling pathway and colorectal cancer. Most of these pathways have been verified associated with CRC initiation and progression by literature exploration above. Here, we continued to discuss some more pathways. Emerging evidence supports the critical roles of the proteoglycans in cancer pathway as the central regulator of cellular homeostasis spanning from early embryonic development to tumour invasion and metastasis. The aberrant activation of this pathway may contribute to tumorigenesis, cancer progression, and treatment resistance [50]. Emerging evidence has supported the critical roles of HIF-1 signaling pathway in cancer dormancy and cancer metabolism, increasing stemness activity and bringing about cancer initiation and progression [51]. There is considerable evidence that VEGF signaling pathway plays an important role in the vascularization and growth of primary tumors as well as in the formation of metastases. VEGF signaling system has been identified an appropriate target to inhibit tumor angiogenesis and metastases formation in CRC [52]. The PPI network analysis including hub genes identification and module analysis may be useful to promulgate the molecular mechanisms of miR-29 family underlying colorectal carcinogenesis.

Our study had several important strengths. Firstly, we carried out a relatively thorough systematic search and applied a comprehensive analytical approach to assess the biomarker values of miR-29 in CRC patients, indicating the widespread influence of miR-29 family on risk, recurrence, metastasis and survival outcome in CRC. Secondly, we explored the diagnostic power of combination biomarkers based on miR-29 and revealed that the miRNA combination biomarkers are more effective than individual miR-29, which may provide new path for progress in clinical practice. Thirdly, we applied an integrative bioinformatics analysis to explore the function of miR-29 family at the systems biology level and to elucidate the reason why miR-29 family members possess such biomarker characteristics. Finally, several interesting results arose from our study, which established a foundation for future observational cohorts and clinical trials.

Although the present study revealed that miR-29 family could become a valuable biomarker for CRC patients, several limitations of this study should be acknowledged. To begin with, some detailed information about the basic characteristics of included studies was not available; thus we could not deal with all the data in a consistent manner. Next, due to limited number of studies about some variables such as races included in the present study, it was unable to conduct subgroup analyses for all types of variables. Moreover, considering lack of sufficient studies associated with the evidence synthesis for the recurrence, metastasis and survival outcome prediction value of miR-29 in CRC; it could not provide strong evidence for the relation between miR-29 family and these clinical observation indexes of CRC.

For the future prospective, several points we can do to optimize the miR-29 assay and promote its application to clinical practice. First, standard cut-offs or thresholds, and detection methods should reach a consensus among different laboratories. Second, since serum miR-29 may be more powerful in detecting CRC compared with plasma miR-29, serum should be considered as a better matrix for further detection. Third, given the superior diagnostic properties of combination biomarkers than single biomarker, more efforts should be devoted to answering the question which and how many miRNAs should be combined with miR-29 to increase the diagnostic performance. Finally, to further develop better diagnostic and prognostic models with higher discriminative capacity, more prospective well-designed randomized controlled studies are required.

## Conclusion

Taken together, our results indicated that miR-29 family had a fine predictive role for the risk, recurrence, metastasis and survival outcome in CRC. In addition, it may be speculated that combining miR-29 and other miRNAs could perform better for clinical application than individual miR-29. Moreover, the integrated bioinformatics findings may be helpful to illuminate the molecular mechanisms underlying colorectal carcinogenesis and to underscore the potential of the miR-29 as novel biomarkers for CRC. However, further large prospective studies are required to explore the biomarker roles of miR-29 family.

## Data Availability

The data supporting the conclusions of this article is within the article.

## Abbreviations

CRC: colorectal cancer
AUC: area under the curve
TP: true positive
FP: false positive
FN: false negative
TN: true negative
PLR: positive likelihood ratios
NLR: negative likelihood ratios
DOR: diagnostic odds ratio
CI: confidence interval
SROC: summary receiver operator characteristic
QUADAS-2: quality assessment of diagnostic accuracy studies 2
GO: gene ontology
KEGG: Kyoto Encyclopedia of Genes and Genomes
PPI: protein–protein interaction
DAVID: database for annotation, visualization, and integrated discovery
STRING: search tool for the retrieval of interacting genes
MCODE: molecular complex detection
BP: biological process
CC: cellular component
MF: molecular function
qPCR: quantitative real-time PCR
